# Experimental analysis of recommended corneal incision sizes in cataract surgery using 13 intraocular lens injector systems

**DOI:** 10.1038/s41598-023-29497-w

**Published:** 2023-02-15

**Authors:** Maximilian Friedrich, Gerd U. Auffarth, Patrick R. Merz

**Affiliations:** David J Apple Center for Vision Research, Department of Ophthalmology, University Hospital Heidelberg, Im Neuenheimer Feld 400, 69120 Heidelberg, Germany

**Keywords:** Lens diseases, Refractive errors, Translational research

## Abstract

Smaller corneal incisions in cataract surgery are linked with a better visual outcome and less frequent postoperative endophthalmitis. The insertion of intraocular lens (IOL) injector systems into the anterior chamber of the eye to implant an IOL is associated with incision enlargement (IE) impeding these positive effects. The aim of this study was to compare manufacturers’ recommended incision sizes (IS) of 13 different intraocular lens injector systems in regard of intraoperative IE and postoperative IS. In total, 499 corneal incisions in ex vivo porcine eyes were analyzed. The preoperative ISs depended on the recommended IS of the examined injector system. The IS was measured right before and after IOL injector insertion with an incision gauge set. There was intraoperative IE in 87% of the incisions with a mean IE of 0.26 ± 0.18 mm. IE was often significantly larger in small IS compared to larger IS concerning an injector system (P < 0.05). Five injector systems needed to have a significantly larger IS than the manufacturers’ recommended IS with an average difference of 0.3 mm when applying study criteria (P < 0.05). Thus, the present study shows that IS recommendations require to be critically analyzed by ophthalmic surgeons to enable evidence-based practice.

## Introduction

Clear corneal incisions (CCI) are a standard method to access the anterior chamber during cataract surgery. In recent decades, these CCIs were subject to intensive research which showed that a smaller incision is desirable as it is associated with less surgically induced astigmatism, less aqueous leakage, less postoperative endophthalmitis, a smaller focal corneal flattening and a better postoperative UCVA and refractive stabilization^1–5^. These findings led to an evolution in the cataract surgery instruments needing to perform safe and successful cataract surgery through progressively smaller CCIs. Prior to this, common surgical procedures, such as intracapsular cataract extraction and traditional extracapsular cataract extraction, involved making a large incision (> 6 mm) to enable an implantation of rigid IOLs using a forceps. An implantation of rigid lenses was not feasible through small incisions. With the introduction of foldable intraocular lenses, it was possible to develop intraocular lens injector systems that could facilitate surgery through mini-incisions (2.0–2.4 mm) and micro-incisions (< 2.0 mm). However, in most cases of mini- or micro-incision cataract surgery, the CCI becomes enlarged during surgery, leading to a bigger postoperative incision size^6–8^. These enlarged incision results are primarily assignable to the effects of insertion of the IOL injector systems into the incision^9^. To our knowledge no study yet has analyzed the incision size recommendations provided by the injector system manufacturers in comparison to the results actually achieved and if the smallest postoperative incision size can be actually achieved by using the manufacturers’ preoperative incision size recommendations during cataract surgery.

This issue is further complicated as new IOL implantation techniques have been developed, such as the Into-the-bag-, the Into-the-wound- or the Wound-assisted-technique, which differ regarding the depth of IOL injector insertion. They involve new IOL injector systems which have a cone angle that enlarges the effective cross diameter of the injector on the CCI as the injector is inserted further into the incision^10^. A larger effective nozzle diameter of an IOL injector would induce more stress on the incision margins and is associated with a higher intraoperative incision enlargement^11^. This could lead to varying incision enlargement depending on the implantation technique and the incision size. Hence, it should be analyzed if manufacturers’ recommendations take this into account.

The purpose of this study was to evaluate incision size recommendations of IOL injector manufacturers by using different preoperative incision sizes and then comparing the intraoperative incision enlargement and the postoperative incision size after the insertion of the IOL injector systems into a CCI. Furthermore, three criteria to determine a new study-based incision size recommendation are introduced.

## Methods

### Study population

In this experimental study, 126 ex vivo porcine eyes (*Sus scrofa domesticus*) from a local abattoir (Schradi Frischfleisch GmbH, Mannheim) were included. All eyes were from animals aged between 5 and 7 months. Experiments were performed within 12 h postmortem and the eyes were constantly stored in a wet chamber while being refrigerated to + 3 °C to avoid degenerative changes and preserve tissue integrity. Exclusion criteria were signs of trauma such as iridodialysis, a prolapsed lens or a ruptured bulbus and a damaged or opacified cornea.

The sample size for each preoperative incision size and IOL injector system was calculated with a power of 0.8, a type I error rate of 0.05 and mean values of postoperative incision sizes with a standard deviation of 0.05. Before surgery, all eyes were randomized into 13 test groups and attached orbital tissue was removed. To prevent corneal stress due to fixation during surgery, the porcine eyes were posteriorly embedded in 3%-Agar solution.

### Injector systems

**Figure 1:**
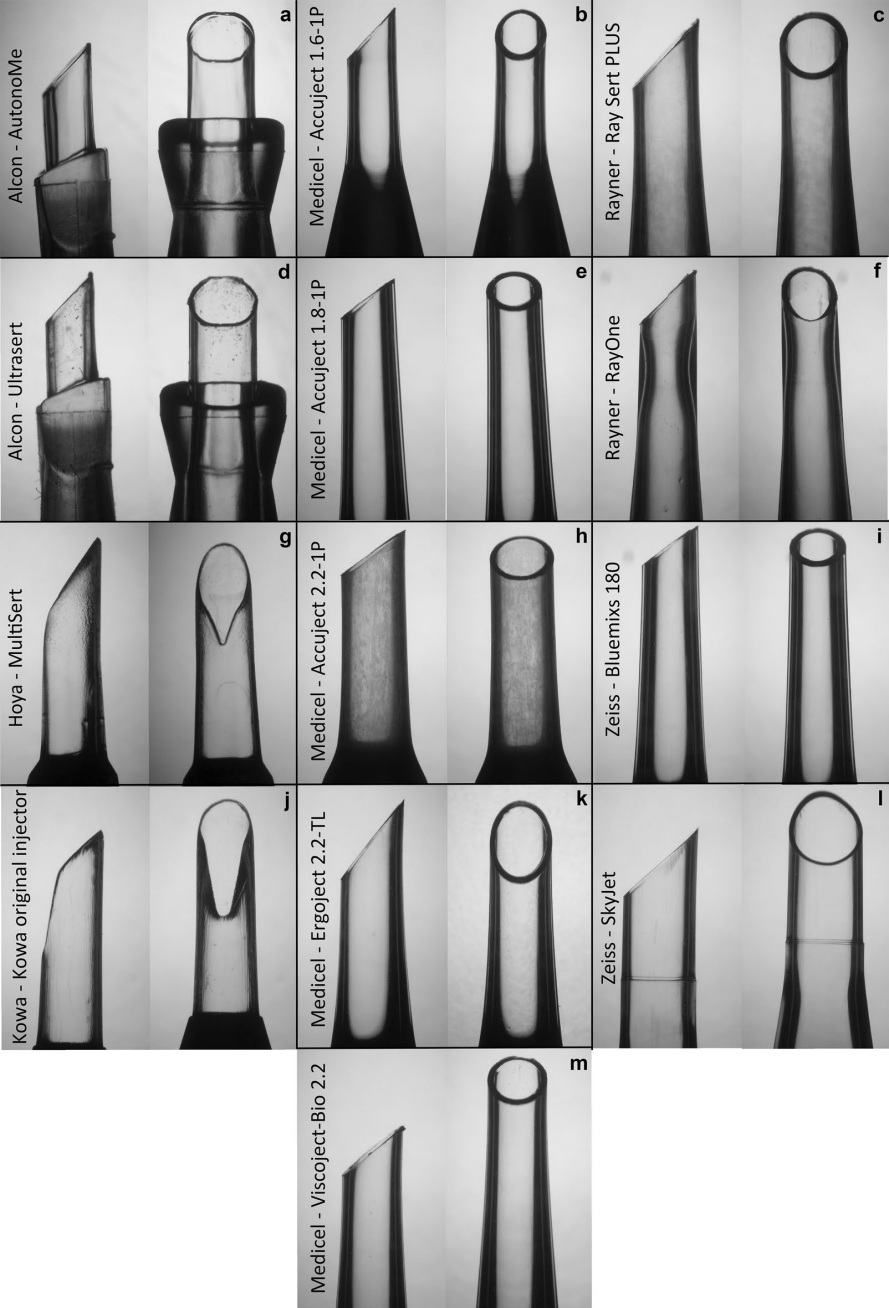
Overview of all microscopic photographed intraocular lens injector systems in a side view (left) and a top view (right). On the left side of each panel is the name of the respective intraocular lens injector system.

As shown in Fig. 1, 126 injectors from 13 injector systems were included in this analysis. Each injector system was photographed under a microscope (Olympus BX50, Olympus Corp.) with an attached camera (Olympus Camedia C-7070 Wide Zoom, Olympus Corp.) to assess morphological differences. The preoperative incision size recommendations, including any recommended IOL implantation techniques, were obtained from the injector package inserts or the manufacturer company websites^12–19^.

### Surgical procedure

**Figure 2:**
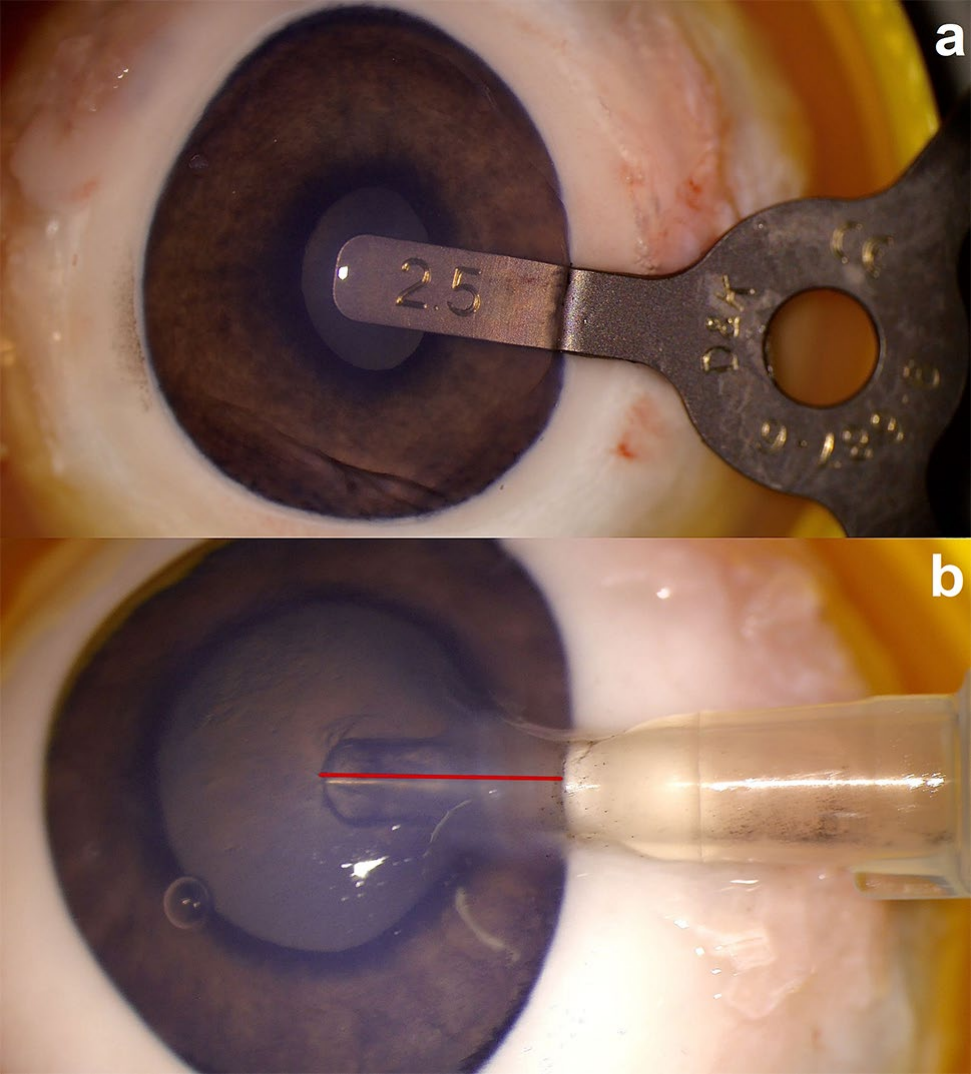
Measurement of surgical parameters. Top: measurement of preoperative incision size of a clear corneal incision using an incision gauge set. Bottom: measurement of intraocular lens injector insertion depth into a clear corneal incision using Image J Software (version 1.52a, NIH).

All surgeries were performed by the same surgeon (M.F.). Each embedded porcine eye was humidified with balanced salt solution during cataract surgery. A triplane self-sealing rectangular CCI was created with a stainless-steel slit knife (Mani Inc.), which varied from 1.8 to 2.8 mm in size. Anterior chamber was filled with the ophthalmic viscosurgical device hydroxypropyl-methylcellulose 2.0% (Pe-Ha-Visco 2.0%, Albomed GmbH) before every IOL injector insertion. Then, the preoperative incision size was measured using a DK incision gauge set (Duckworth & Kent Ltd.), which measures from 1.0 to 3.0 mm with an interval of 0.1 mm. All measurements were conducted from highest to lowest incision gauge size, to prevent a procedural enlargement of the incisions due to insertion of the gauge. Each IOL injector was prepared for surgery according to their respective instructions for use. If an IOL was available, it was loaded into the cartridge to ensure the injector was in the actual operating condition. The prepared injector was then inserted into the incision with an insertion depth comparable to the Into-the-bag IOL implantation technique. The Into-the-Bag IOL implantation technique was used for each IOL injector model regardless of the respective recommended implantation technique to promote comparability between the various IOL injector models in this study. Insertion depth was documented using a camera (DMC-G6, Panasonic Corp.) attached to a microscope (Leica M220, Leica Microsystems GmbH). After calibrating each photograph with a scale on incision level, insertion depth was measured using Image J Software (version 1.52a, NIH, Fig. 2). Where no IOL was available, the IOL injectors were inserted into the incision in a fashion comparable to the Into-the-bag IOL implantation technique, but without implanting an IOL.

**Figure 3:**
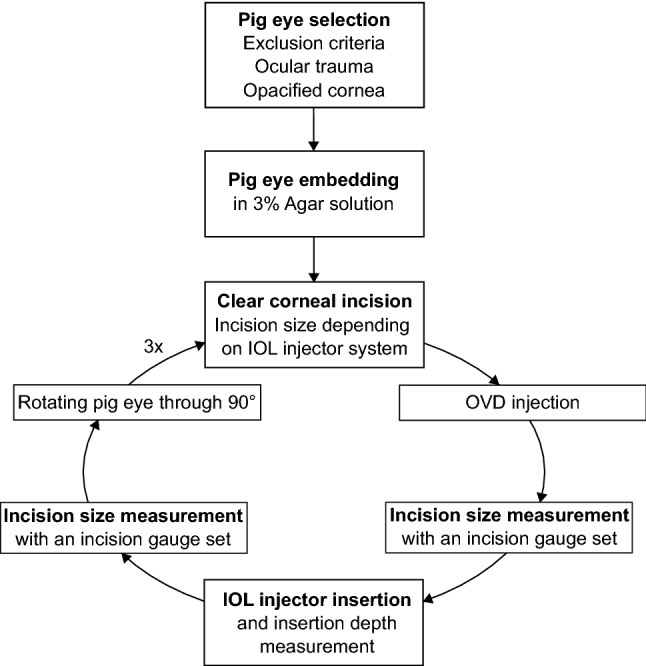
Visualization of the surgical process. *OVD* ophthalmic viscosurgical device, *IOL* intraocular lens.

After IOL injector insertion and removal, the postoperative incision size was again measured with previously mentioned DK incision gauge set to measure intraoperative wound stretching (Fig. 2). The incision was not intentionally extended using a blade at any point. The porcine eye was then rotated through 90° around the vertical axis and a new triplane self-sealing rectangular CCI was created with a different incision size as compared to the first incision in that eye. Subsequently, the complete process of the previously mentioned insertion and measurement steps were performed again. After completion of the second round of tests, the porcine eye was twice again rotated through 90° and underwent surgery for a third and fourth time with different incision sizes. If an IOL was available, it was only implanted into the anterior chamber through the fourth incision of the porcine eye to minimize any change or damage to the injector nozzle, which could have influenced the postoperative outcomes if used through one of the earlier incisions. The process is visualized in Fig. 3. For one IOL injector system, the *SkyJet* (Carl Zeiss Meditec AG), only three incisions per eye were made as the enlarged postoperative incisions reached the scale limit of 3.0 mm of the incision gauge set. In summary, four incisions were analyzed in almost all porcine eyes which adds up to a total of 499 analyzed incisions.

### Study-based incision size

To obtain a study-based preoperative incision size recommendation, the following criteria were considered:

Primarily, the recommended incision size should not pose an unreasonable surgical challenge. This should ensure safety and standardization of cataract surgery.

Secondly, the recommended preoperative incision size should lead to the smallest achievable postoperative incision size to benefit of the advantages of mini- and micro-incision cataract surgery.

Thirdly, the recommended incision size should lead to the least intraoperative incision enlargement. This enlargement is evoked by stress onto the incision margins which can lead to uncontrolled tear and other unknown consequences^11^.

After the surgeries, the resulting postoperative incision sizes, subdivided by preoperative incision size were statistically compared to each other for each IOL injector system. If a preoperative incision size led to a significantly larger postoperative incision size, it was excluded from further analysis.

All remaining preoperative incision sizes were statistically compared in terms of the respective intraoperative incision enlargement for each IOL injector system. If a preoperative incision size led to a significantly larger intraoperative incision enlargement, it was excluded from the analysis. The remaining preoperative incision sizes were reported as study-based incision sizes.

### Data analysis

The statistical analysis was performed using SPSS for Windows (Version 28, SPSS Inc.). Intraoperative incision enlargement was calculated by subtracting the measured preoperative incision size from the measured postoperative incision size for each incision. Preoperative incision sizes were compared in respect of postoperative incision size and intraoperative incision enlargement using one-way analysis of variance (ANOVA) and a Games–Howell post hoc test, considering inequal variances and inequal sample sizes. For each IOL injector system a family-wise type I error controlled at 0.05 was reached with Bonferroni adjustments. Differences of injector insertion depth with and without IOL implantation was evaluated by using an independent *t* test, where a *P* value less than 0.05 was considered statistically significant.

### Conference presentation

Part of the results in this paper were presented at the 39th Congress of the European Society of Cataract & Refractive Surgeons, held in Amsterdam, The Netherlands, and online, in October 2021.

## Results

### Injector systems

The injector nozzles of the included injector systems are shown in Fig. 1. *AutonoMe* and *Ultrasert* (both Alcon Inc.) had *insert shields*, which should prevent a deep insertion of the injector into the incision. IOL injector system *Accuject 1.6-1P* (Medicel AG) had a steep cone angle after 4.28 mm ± 0.1 mm. *MultiSert* (Hoya Medical Singapore Ltd.) and Kowa original injector (Kowa Company Ltd.) had a notch in the inferior nozzle wall.

For all IOL injector systems a recommended preoperative incision size could be obtained. As shown in Table 1, five injector systems (38%) had a specific recommendation for the Into-the-bag IOL implantation technique, two injector systems (15%) had only a specific recommendation for another IOL implantation technique (wound assisted and wound-in), and six injector systems (46%) had an incision size recommendation without any IOL implantation technique specification. The smallest and largest recommended incision size was 1.8 mm (*Bluemixs 180*, Carl Zeiss Meditec AG) and 2.8 mm (*SkyJet*, Carl Zeiss Meditec AG), respectively. The smallest recommended incision size for the Into-the-bag IOL implantation technique was 2.0 mm (*Accuject 1.6-1P*, Medicel AG).

**Table 1:** Preoperative incision size recommendations of intraocular lens injector systems.

Injector system	Manufacturer	N	Manufacturer recommendation		Study based incision size^a^ (mm)
			IOL implantation technique	Preoperative incision size (mm)	
AuotonoMe	Alcon Inc	12	N/A	2.2*	2.4*
Ultrasert	Alcon Inc	10	N/A	2.2	2.2–2.4
MultiSert	Hoya Medical Singapore Ltd	10	N/A	2.2	2.2–2.3
Kowa original injector	Kowa Company Ltd	10	N/A	2.4	2.3–2.4
Accuject 1.6-1P	Medicel AG	5	Into-the-bag	2.0	1.9–2.1
Accuject 1.8-1P	Medicel AG	12	Into-the-bag	2.2*	2.3–2.4*
Accuject 2.2-1P	Medicel AG	10	Into-the-bag	2.5	2.4–2.5
Ergoject 2.2-TL	Medicel AG	5	Into-the-bag	2.5	2.4–2.5
Viscoject-Bio 2.2	Medicel AG	10	Into-the-bag	2.5	2.4–2.8
Ray *Sert* PLUS	Rayner Intraocular Lenses Ltd	19	Wound-assisted	2.2*	2.6*^b^
RayOne	Rayner Intraocular Lenses Ltd	8	Wound-in	2.2*	2.6*
Bluemixs 180	Carl Zeiss Meditec AG	10	N/A	1.8*	2.2–2.3*
SkyJet	Carl Zeiss Meditec AG	5	N/A	2.8	2.8^b^

*IOL* intraocular lens. ^a^Using an Into-the-bag IOL implantation technique. ^b^Possibly undersized due to reaching the scale limit of 3.0 mm. *Discrepancy between the manufacturers’ preoperative incision size recommendation and the study-based preoperative incision size.

### Insertion depth

In total, 499 incisions in 126 porcine eyes were examined. The results of the insertion depth measurement are shown in Table 2. The injector systems Accuject 1.6-1P, AutonoMe and Ultrasert were significantly less deep inserted into incisions compared to the other injector systems (P < 0.001). Mean insertion depth of the other injector systems ranged from 5.5 to 6.64 mm with a mean standard deviation of 0.56 mm. There was no statistically significant difference in insertion depth between injector insertions with IOL implantation and without IOL implantation (P = 0.23).

**Table 2:** Insertion depth of intraocular lens injector systems into a clear corneal incision.

Injector system	Manufacturer	Insertion depth (mm)		
		Mean	SD	95% CI
AuotonoMe	Alcon Inc	2.75	0.18	2.69–2.81
Ultrasert	Alcon Inc	2.62	0.19	2.54–2.70
MultiSert	Hoya Medical Singapore Ltd	5.53	0.37	5.41–5.65
Kowa original injector	Kowa Company Ltd	6.04	0.33	5.93–6.15
Accuject 1.6-1P	Medicel AG	4.54	0.29	4.38–4.70
Accuject 1.8-1P	Medicel AG	6.00	0.55	5.83–6.17
Accuject 2.2-1P	Medicel AG	6.06	0.43	5.92–6.20
Ergoject 2.2-TL	Medicel AG	5.50	0.45	5.25–5.74
Viscoject-Bio 2.2	Medicel AG	5.57	0.50	5.34–5.80
Ray *Sert* PLUS	Rayner Intraocular Lenses Ltd	6.33	0.63	6.17–6.48
RayOne	Rayner Intraocular Lenses Ltd	6.30	0.64	6.05–6.55
Bluemixs 180	Carl Zeiss Meditec AG	6.20	0.51	6.04–6.37
SkyJet	Carl Zeiss Meditec AG	6.64	1.23	5.96–7.33

### Study-based incision size

**Figure 4:**
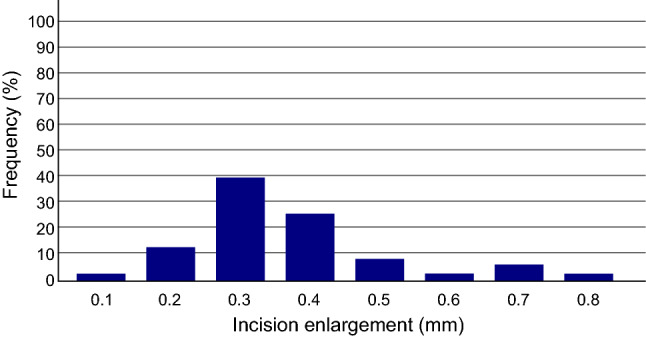
Frequency of intraoperative incision enlargement due to intraocular lens injector insertion into a clear corneal incision while using the manufacturers’ recommended incision sizes.

In 433 of 499 incision (87%) intraoperative incision enlargement was observed. The median and maximum intraoperative incision enlargement was 0.2 mm and 0.8 mm, respectively. There was no IOL injector system that did not lead to some intraoperative incision enlargement. Figure 4 shows that the manufacturers’ recommended incision sizes led to all incisions being intraoperatively enlarged with a mean increase of 0.37 mm ± 0.15 mm. The maximum incision enlargement was 0.8 mm in a preoperatively 2.2 mm sized incision, which equals an enlargement of 36.36%.

**Figure 5:**
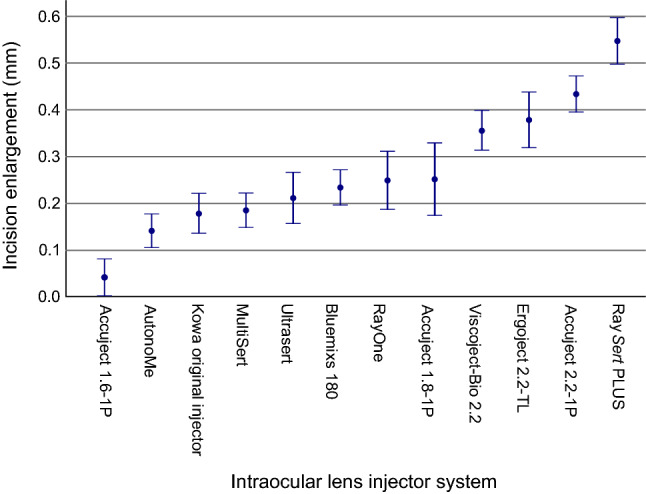
Mean intraoperative incision enlargement in a 2.4 mm sized clear corneal incision due to intraocular lens injector insertion. The error bar represents a 95% confidence interval.

The differences of intraoperative incision enlargement between the IOL injector systems while using the same preoperative incision size of 2.4 mm are visualized in Fig. 5. A preoperative incision size of 2.4 mm is identical or greater than 9 of the 13 manufacturers’ incision size recommendations. However, nearly all IOL injector models enlarged the incision intraoperatively. The IOL injector system *SkyJet* (Carl Zeiss Meditec AG) could not fit into a 2.4 mm sized incision and was therefore excluded from Fig. 5.

Whether an IOL injector was inserted with or without an IOL implantation did not have a significant effect on the intraoperative incision enlargement (P = 0.34) or the postoperative incision size (P = 0.13).

**Figure 6:**
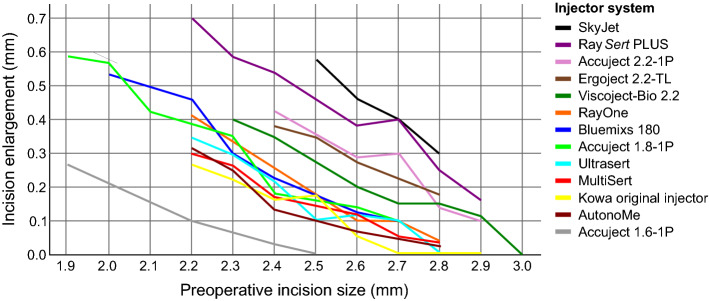
Interpolated means of intraoperative incision enlargement in varying preoperative incision sizes due to intraocular lens injector insertion.

Figure 6 shows the intraoperative incision enlargement in regard of the preoperative incision size. Smaller preoperative incisions tend to have a higher incision enlargement compared to large preoperative incisions. The smallest postoperative incision size of 2.1 mm was achieved by using an *Accuject 1.6-1P* in a preoperatively 1.9 mm sized incision. *SkyJet* injector systems lead to the highest incision enlargement in large preoperative incisions over 2.5 mm.

The study-based incision size that were obtained through this analysis are presented in Table 1. The study-based incision sizes ranged from 1.9 to 2.8 mm and were presented as a range if there was not a statistically significant difference in the postoperative incision size and the intraoperative incision enlargement when comparing different preoperative incision sizes.

In total, thirteen IOL injector systems from six manufacturers were included in this analysis and thirteen (100%) incision size recommendations of the manufacturers could be obtained. Compared to the manufacturers’ incision size recommendations, five (38%) study-based incision sizes were larger. The difference of study-based incision sizes to the manufacturers’ recommendation of the injector systems *AutonoMe*, *Accuject 1.8-1P*, *Ray Sert PLUS*, *RayOne* and *Bluemixs 180* was 0.2 mm, 0.1 mm, 0.4 mm, 0.4 mm, and 0.4 mm, respectively. Of these five IOL injector systems, one (20%) had a specific incision size recommendation for the Into-the-bag IOL implantation technique, two (40%) had only a specific incision size recommendation for a different IOL implantation technique and two (40%) had an incision size recommendation without specifying an IOL implantation technique. The other eight (62%) recommended incision sizes of the manufacturers were equal to or in the range of the respective study-based incision size. The IOL injector systems *Ray Sert PLUS* (Rayner Intraocular Lenses Ltd.) and *SkyJet* (Carl Zeiss Meditec AG) reached the 3.0 mm limit of the incision gauge set. Because of this, it was not possible to precisely measure the maximum intraoperative incision enlargement and the maximum postoperative incision size for these IOL injector systems in this study.

## Discussion

Clear corneal incisions often enlarge during cataract surgery which can mainly be attributed to insertion of an IOL injector system into the CCI^9^. Therefore, incision size recommendations of IOL injector manufacturers should be precise and study-based to enable a safe and controlled surgery. To our knowledge, this was the first study which compared manufacturers’ preoperative incision size recommendations to a transparent preoperative incision size analysis, and measured insertion depth of IOL injector systems into a CCI.

The lack of incision size recommendations linked with a specific IOL implantation technique, as shown in Table 1, impedes an informed incision size choice for a cataract surgeon. Without a specified recommendation, an unnecessarily large preoperative incision size would lead to a needlessly large postoperative incision size and therefore a worse clinical outcome. On the contrary, an unnecessarily small preoperative incision size may hinder an IOL injector insertion, and a high force would be needed to insert the IOL injector into the CCI.

Additionally, this study showed that the usage of an undersized preoperative incision is associated with a bigger intraoperative incision enlargement. Previous studies have shown that a smaller preoperative incision leads to more intraoperative incision enlargement. For example, Oshika et al., Arboleda et al. and Zhang et al. showed that a smaller preoperative incision size leads to a larger intraoperative incision enlargement and is not always associated with a smaller postoperative incision size^10,20,21^. In accordance with these results, Yildirim et al. found a larger preoperative incision size to have less intraoperative incision enlargement^22^. However, a bigger intraoperative incision enlargement is associated with a higher surgically induced astigmatism^1,22^.

These findings suggest that it is not sufficient for an incision size recommendation to be solely based on the minimal preoperative incision size to perform surgery on or the smallest achievable postoperative incision size. Several variables need to be considered to find the optimal incision size recommendation. Yet, IOL injector manufacturers did not provide any studies to show how their incision size recommendation was determined and consequently the applied criteria remain unknown. Other studies comparing IOL injector systems focused on the postoperative outcome and the intraoperative incision enlargement while using one constant preoperative incision size for all IOL injector models^23–25^.

In this study, a transparent approach to determine study-based preoperative incision sizes for 13 IOL injector systems was established. As shown in Table 1, five discrepancies between the manufacturers’ recommendations and the study based preoperative incision sizes were found. This novel approach included the factors of surgical challenge, postoperative incision size and intraoperative incision size enlargement in that order. After considering all factors, finally a range of preoperative incision sizes with a feasible surgical challenge and resulting in the smallest possible postoperative incision size while having a small intraoperative incision enlargement could be obtained. Other factors, such as the architecture of the incision or the corneal thickness, could also influence the choice of the best preoperative incision size but these factors were not included in this analysis.

The five discrepancies between the manufacturers’ preoperative incision size recommendations and the study based preoperative incision sizes found in this study should alert surgeons that the manufacturers’ recommendations are currently lacking evidence and have not been obtained independently through transparent criteria studies. The incision size recommendations could therefore be influenced by marketing aspects and competition between manufacturers. Hence, the manufacturers’ recommendation should be used with care.

There were three IOL injector systems which had a morphology preventing a deep insertion into the incision. To show differences in insertion depth for these models, insertion depth was measured for the first time in cataract research. Insertion depth could be an important variable influencing the smallest achievable postoperative incision size. As previous studies pointed out, a larger cross diameter of the IOL injector nozzle is associated with a larger postoperative incision size^10,21,26^. As Arboleda et al. presented, in many IOL injectors there is a cone angle^10^. Therefore, a deeper IOL injector insertion could lead to a bigger cross diameter effecting on the CCI and consequential to a larger postoperative incision size. A restriction of insertion depth by using an insertion shield or an IOL injector system with a neglectable cone angle could solve this issue.

Comparing the intraoperative incision enlargement in a 2.4 mm sized incision between all included IOL injectors in Fig. 5, it is visible that most of the IOL injectors cause a substantial intraoperative incision enlargement. These findings are remarkable since 9 of the 13 manufacturers’ incision size recommendations are identical or smaller than the pictured 2.4 mm sized incisions. Only the IOL injector *Accuject 1.6-1P* (Medicel AG) could implant an IOL through a 2.4 mm sized incision with a mean intraoperative incision enlargement of less than 0.1 mm. This should raise awareness to the nearly ubiquitous enlargement of the incision during the use of IOL injector systems in commonly sized corneal incisions.

Furthermore, there was no difference in postoperative incision size and intraoperative incision enlargement comparing incisions with an IOL implantation to incisions without an IOL implantation (P > 0.05). This contradicts previous findings, where the passage of an IOL was associated with a higher intraoperative incision enlargement^26,27^. An explanation for this phenomenon could be the variety of IOL injector nozzles included in this study. Possibly, only a part of IOL injector nozzles enlarge during the passage of an IOL while other IOL injector nozzles maintain a more constant diameter. Further sophisticated studies are needed to investigate the behavior of IOL injector nozzles during IOL passage.

There were several limitations in this study. Ex vivo porcine eyes have a thicker cornea than humans and were used as a replacement as is customary in ophthalmologic laboratory studies^28^. Additionally, four clear corneal incisions per eye were examined and only in one of four incisions an IOL was implanted. This was addressed by comparing the relevant outcome parameters postoperative incision size and intraoperative incision enlargement to a standard cataract surgery with one main incision and finding no statistically significant differences (P = 0.57).

The intraocular pressure during cataract surgery was not measured and could therefore vary between IOL injector insertions and between individual pig eyes. To enhance the comparability between all IOL injector insertions, we filled the anterior chamber with an ophthalmic viscosurgical device before every IOL injector insertion.

The injection of IOLs into the anterior chamber could have influenced the insertion angle and insertion depth of the IOL injectors compared to regular cataract surgery and therefore the forces applied to the incision margins. While the insertion depth was measured using the above-mentioned novel technique to maintain a comparable surgical use between the IOL injector systems, the insertion angle was not measured. An implantation posteriorly into the capsular bag could lead to a steeper insertion angle compared to a more uniplanar implantation into the anterior chamber. However, a uniplanar insertion should lead to a reduced stress onto the incision margins due to the reduced manipulation of the incision. If there is intraoperative incision enlargement while using a potentially less traumatic insertion technique, this should highlight the necessity of evaluation of incision size recommendations.

Lastly, in this study only incision enlargement attributable to insertion of an IOL injector system was investigated. Incision enlargement due to other aspects of cataract surgery, such as phaco tip movement or implantation of IOLs with varying diopters, were not assessed.

This study compared 126 IOL injectors of 13 IOL injector systems regarding their preoperative incision size recommendations. We found that despite using specific incision size recommendations, there was always an intraoperative incision enlargement in all thirteen injector systems, and five (46%) manufacturers’ recommendations did not specify an IOL implantation technique. Therefore, based on the results of our experiments, a transparent algorithm for finding a preoperative incision size recommendation, considering the factors surgical challenge, postoperative incision size and intraoperative incision enlargement, was developed.

Another key result is that we can recommend the use of five study-based preoperative incision sizes which differ from the manufacturers’ recommendations. This data should be included into the surgeon’s decision process regarding the choice of an adequate preoperative incision size to reduce intraoperative incision enlargement. Additionally, the insertion depth of an IOL injector system into a CCI was measured and this could be used as a parameter in future studies to compare IOL implantation techniques or to explain variance of postoperative incision size. In future studies, the clinical outcome between large preoperative incision sizes with small intraoperative incision enlargement and small preoperative incision sizes with large intraoperative incision enlargement should be compared.

## Data Availability

The data that support the findings of this study are available from the corresponding authors upon reasonable request.
